# Renal Lesions and Nephrotoxicity: Contemporary Challenges and Future Directions

**DOI:** 10.3390/ijms24087015

**Published:** 2023-04-10

**Authors:** Egor Plotnikov

**Affiliations:** A.N. Belozersky Institute of Physico-Chemical Biology, Lomonosov Moscow State University, 119991 Moscow, Russia; plotnikov@belozersky.msu.ru

Renal lesions and nephrotoxicity are major challenges for researchers and patients alike. They have a variety of causes and mechanisms, including metabolic disorders, chronic inflammatory conditions, cardiorenal syndrome, infectious diseases, and aging. Acute and chronic kidney diseases are common problems that affect millions of people worldwide. With this in mind, we need to ask ourselves how much we really understand about the pathogenesis and treatment of kidney diseases.

One of the most important causes of renal damage is systemic metabolic dysfunction, such as obesity, metabolic syndrome, and diabetes. It plays a pivotal role in chronic kidney disease (CKD), and CKD, in turn, can exacerbate metabolic disorders, creating a vicious cycle of disease progression. Lifestyle-related factors, such as diet, obesity, and hypertension have a detrimental impact on our kidneys, so we need to focus our efforts on prevention strategies for early-stage kidney disease and uncovering the mechanisms underlying kidney injury in diabetes and associated disorders. Diabetic nephropathy remains the most common complication of diabetes, which generates relentless interest of researchers regarding the study of this pathology. It is characterized by progressive damage to the renal glomeruli and tubules, leading to the development of CKD, as well as further end-stage renal disease. In this regard, there is a need for non-invasive and sensitive markers for early detection of nephropathy in diabetes. Park and colleagues found that pyruvate kinase M2 was excreted in the urine of rats with acute kidney injury [1], and it can be used as a biomarker for the diagnosis of DN in diabetic patients, as it was also highly detected in the urine of diabetic patients.

Moreover, diabetes may be a secondary complication in kidney transplantation. Its development is associated with immunosuppressive therapy, and this may affect the transplant recipient. This can result in poor graft function, increased risk of infections, and other complications. Diabetes is related to some of the most widely studied renal complications globally. Jehn and coauthors investigated the impact of tacrolimus metabolism [2], which is associated with increased nephrotoxicity and unfavorable outcomes in kidney transplantation, with an increase in the risk for post-transplant diabetes mellitus. Data from patients and experimental modeling of fast and slow tacrolimus metabolism kinetics in cultured insulin-producing pancreatic cells showed that fast tacrolimus metabolism is not associated with an increased incidence of diabetes, either in vitro or in vivo.

Cardiorenal syndrome is another pathology that requires a holistic approach. The kidney and heart are closely interconnected, and dysfunction in one organ can lead to dysfunction in the other. Cardiorenal syndrome is a condition that describes the bidirectional relationship between heart and kidney dysfunction. In cardiac arrest, renal ischemia, as well as entire body ischemia, can occur due to decreased blood flow. This can lead to acute kidney injury (AKI), which is associated with poor outcomes. Evidence suggests that clinically significant AKI is rarely associated with a single cardiac arrest case, and it is followed by the return of spontaneous circulation. However, AKI can occur in patients with pre-existing renal impairment or those receiving renin-angiotensin-aldosterone system blockade therapy. To better understand the mechanisms of kidney damage in cardiac arrest, Tsivilika and colleagues performed a study of the morphology and ultrastructural changes in kidney cells in ischemia that accompanies cardiac arrest [3]. It is important to note that the study was performed in pigs because of the size of these animals, and their circulation and renal function are quite similar to those in humans. The authors concluded that acute kidney injury is not the main cause of death after cardiac arrest. However, the available data suggest that kidney damage after cardiac arrest should be considered when predicting the health status of patients.

Regardless of whether we consider chronic or acute kidney pathologies, the loss of kidney functions ultimately depends on the regenerative potential. Although the kidney belongs to postmitotic organs, the presence of a pool of progenitor cells within it, as well as the possibility of dedifferentiation of the epithelium, create the conditions for regeneration, which allows one to reverse loss-of-function. However, as with many cell types in the body, the regenerative potential of the kidney decreases with age. This age-related decline in kidney recovery can limit the therapeutic effectiveness of any nephroprotective approaches. The mechanisms underlying this decline are not yet fully understood, but they may involve cellular senescence, changes in the microenvironment, alterations in signaling pathways, and, finally, reducing the number of progenitors in the kidney. Using genetically modified mice, it was shown that, with increasing age, the number of resident progenitor cells in the kidney decreases, and these cells themselves have a reduced proliferative potential [4]. As a result, these changes have been associated with reduced resistance to damaging factors in cells derived from old mice.

Understanding the mechanisms that control the regenerative potential of kidney progenitor cells will be crucial for the development of effective renal regeneration therapies. Strategies for overcoming the age-related decline in regenerative potential, such as enhancing signals from the microenvironment that promote progenitor cell proliferation, will be critical in developing new clinical interventions, reducing the need for high-risk renal transplantation, and offering the potential for improved quality of life.

Renal pathologies are always closely associated with various diseases, making the kidney a sensitive nexus in a variety of disorders in the organism. Recently, COVID-19 has brought a new set of challenges to the table. The COVID-19 pandemic has highlighted the importance of kidney health, as kidney failure is a common complication of severe COVID-19 infection. The exact mechanisms underlying COVID-19-related kidney injury are not yet fully understood, but they are thought to be related to inflammation and endothelial dysfunction. COVID-19 is also among the causative factors of rhabdomyolysis. Młynarska and colleagues presented an exhaustive review [5] of the relationship between rhabdomyolysis, renal dysfunction, and COVID-19, especially from the perspective of the development of systemic inflammation, which leads to increased kidney damage caused by myoglobin toxicity. The need for timely diagnosis of rhabdomyolysis has been convincingly demonstrated, especially when signs of renal insufficiency have not yet manifested themselves. Possible methods of AKI treatment are also considered in terms of the adequacy of their use and possible side effects in patients with COVID-19 or severe systemic inflammation. The management of COVID-19-related kidney injury requires a multidisciplinary approach and close monitoring of kidney function. The pandemic’s deleterious impact on kidney function requires uncovering the precise mechanisms that lead to renal damage in COVID-19 patients and looking for the most suitable treatment options for critically ill patients.

In conclusion, kidney damage is a complex problem that requires a comprehensive approach to management. In this Special Issue, we have outlined how metabolic disorders, including diabetes, renal ischemia, rhabdomyolisis, and COVID-19, as well as impairment in renal regeneration, are critical areas that require more research and attention. The development of novel therapeutic approaches and strategies for early detection of nephropathies and kidney disease is urgently needed. We must continue our efforts to develop a better understanding of renal injury initiation, progression, and healing.
